# Schwann Cell Autocrine and Paracrine Regulatory Mechanisms, Mediated by Allopregnanolone and BDNF, Modulate PKCε in Peripheral Sensory Neurons

**DOI:** 10.3390/cells9081874

**Published:** 2020-08-11

**Authors:** Veronica Bonalume, Lucia Caffino, Luca F. Castelnovo, Alessandro Faroni, Flavio Giavarini, Sheng Liu, Donatella Caruso, Martin Schmelz, Fabio Fumagalli, Richard W. Carr, Valerio Magnaghi

**Affiliations:** 1 Department of Pharmacological and Biomolecular Sciences, Università degli Studi di Milano, 20133 Milan, Italy; veronica.bonalume@unimi.it (V.B.); lucia.caffino@unimi.it (L.C.); luca.castelnovo@utexas.edu (L.F.C.); flavio.giavarini@unimi.it (F.G.); donatella.caruso@unimi.it; (D.C.); fabio.fumagalli@unimi.it (F.F.); 2 Marine Science Institute, The University of Texas at Austin, 750 Channel View Drive, Port Aransas, TX 78373, USA; 3 Blond McIndoe Laboratories, Division of Cell Matrix Biology and Regenerative Medicine, Faculty of Biology, Medicine and Health, The University of Manchester, Manchester M13 9PL, UK; alessandro.faroni@manchester.ac.uk; 4 Institute of Pharmacology, Heidelberg University, 68167 Mannheim, Germany; sheng.liu@pharma.uni-heidelberg.de; 5 Experimental Pain Research, Medical Faculty Mannheim, Heidelberg University, 68167 Mannheim, Germany; martin.schmelz@medma.uni-heidelberg.de (M.S.); richard.carr@medma.uni-heidelberg.de (R.W.C.)

**Keywords:** neuro-glia interaction, neuropathic pain, hyperalgesia, dorsal root ganglia, neuroactive steroid

## Abstract

Protein kinase type C-ε (PKCε) plays important roles in the sensitization of primary afferent nociceptors, such as ion channel phosphorylation, that in turn promotes mechanical hyperalgesia and pain chronification. In these neurons, PKCε is modulated through the local release of mediators by the surrounding Schwann cells (SCs). The progesterone metabolite allopregnanolone (ALLO) is endogenously synthesized by SCs, whereas it has proven to be a crucial mediator of neuron-glia interaction in peripheral nerve fibers. Biomolecular and pharmacological studies on rat primary SCs and dorsal root ganglia (DRG) neuronal cultures were aimed at investigating the hypothesis that ALLO modulates neuronal PKCε, playing a role in peripheral nociception. We found that SCs tonically release ALLO, which, in turn, autocrinally upregulated the synthesis of the growth factor brain-derived neurotrophic factor (BDNF). Subsequently, glial BDNF paracrinally activates PKCε via trkB in DRG sensory neurons. Herein, we report a novel mechanism of SCs-neuron cross-talk in the peripheral nervous system, highlighting a key role of ALLO and BDNF in nociceptor sensitization. These findings emphasize promising targets for inhibiting the development and chronification of neuropathic pain.

## 1. Introduction

Schwann cells (SCs) are the main glial cells of the peripheral nervous system (PNS). Myelinating SCs form the myelin sheath, which electrically isolates axons and allows saltatory conduction of the action potentials. Non-myelinating SCs surround unmyelinated fibers to form Remak bundles, where SCs ensheath multiple small-caliber axons, isolating one from another [1,2]. The majority of unmyelinated axons serve sensory function associated with thermoception and nociception. Besides these structural purposes, SCs cross-interact with neurons, specifically with whole axons, regulating their physiological functions. A plethora of mediators is produced and/or released by SCs, thus regulating the neuron-glial interaction, including neuropeptides, cytokines, growth factors, integrins, neuregulins, neurotransmitters and neuroactive steroids [3,4,5,6,7].

The progesterone metabolite 5α-pregnan-3α-ol-20-one, named tetrahydroprogesterone or allopregnanolone (ALLO), is the most important neuroactive steroid, targeting both neurons and glial cells in the PNS [8]. ALLO is produced by SCs, since these cells possess the biosynthetic enzymatic complex [9,10,11]. In the PNS, ALLO participates in the control of myelination, nerve regeneration [9,12,13,14] and also nociception [15]. ALLO capacity to modulate nociceptive pathways has long been ascribed to its allosteric activation of the GABA type A (GABA_A_) receptor, thus potentiating its inhibitory role, at least in central nervous system (CNS) synapses [16].

The ε isoform of protein kinases C (PKCε) possesses several roles in the function of the nervous system [17,18], and in particular in pain modulation. Rather than simply inducing an acute increase of nociceptor excitability, PKCε is crucial in establishing a long-term sensitization, also termed hyperalgesic priming [19,20]. According to this view, PKCε acts by phosphorylating several ion channels, to increase neuronal excitability. Indeed, PKCε lowers the heat activation threshold of TRPV1 [21,22], decreases GABA_A_ receptor inhibitory effect [23,24,25] and upregulates Na_v_1.8 expression [26]. Importantly, PKCε reduces GABA_A_ receptor sensitivity to ALLO [27]. Very preliminary data showed that ALLO upregulates PKCε expression in peripheral neurons via SC activation [28], although the mechanism behind this modulation is still unclear. Altogether, the cross interaction between ALLO, GABA_A_ and PKCε rises the hypothesis of a complex modulation of peripheral pain pathways.

Based on these lines of evidence we tested the hypothesis that PKCε in peripheral sensory neurons may be locally modulated by SCs. Using a combination of biomolecular and pharmacological approaches we studied rat primary SCs and dorsal root ganglia (DRG) neurons in vitro, investigating whether PKCε in neurons is regulated by ALLO and/or other factors released by SCs. Our data highlight a novel mechanism through which peripheral glial-to-neuron cross-talk autocrinally and paracrinally controls PKCε and likely neuropathic pain onset and chronification.

## 2. Material and Methods

### 2.1. Animals

All experiments involved newborn and adult male Sprague–Dawley (Charles River) rats and were performed in accordance with current European rules concerning care and use of animals (Council Directive 2010/63/EU of the European parliament and the Council of 22 September 2010 on the protection of animal used for scientific purposes) and according to 3R’s guidelines. Animal authorization was: project number 478/2015-PR, approved the 3rd June 2015.

### 2.2. Sensory Neurons and SCs Primary Cultures

Primary sensory neurons were obtained from DRG, as previously described [14,29], with minor modification. Briefly, DRG were harvested and dissociated for 40 min in Ham’s F12 medium (Life Technologies Italia, Monza, Italy), containing 0.125% (*w/v*) collagenase Type IV (Worthington Biochemical, Lakewood, NJ, USA), followed by 30 min digestion with 0.25% (*w/v*) trypsin (Worthington Biochemical) and filtration with 100 μm membrane (BD Biosciences, Milan, Italy). Cells were suspended in Ham’s F12 and purified on a gradient of 20% (*w/v*) bovine serum albumin (BSA; Sigma-Aldrich, Milan, Italy). The dissociated neurons were suspended in Bottenstein and Sato’s medium with minor changes [BSM; F12 medium plus N2 (100 μM putrescine, 30 nM sodium selenite, 20 nM progesterone, 0.1 mg/mL BSA, 1.3 mM transferrin, 10 pM insulin) and nerve growth factor 50 ng/mL, all Sigma-Aldrich] plus 10 µM arabinoside C (AraC; Sigma-Aldrich). A cell suspension (1.5 × 10^4^) of neurons was seeded on a 35 mm petri dish coated with 2 µg/mL poli-L-lysine and 2 µg/mL laminin (Sigma-Aldrich). DRG neurons were characterized by immunofluorescence for neurofilament of 200 kDa (NF200, Sigma-Aldrich), as described [14,29]. SCs cultures were obtained as previously described [14,30]. Sciatic nerves were digested with 1% collagenase and 0.25% trypsin (Sigma-Aldrich), then mechanically dissociated, filtered through a 100 µm filter (BD Biosciences) and centrifuged 5 min at 900 rpm. Pellets were suspended in Dulbecco’s modified Eagle’s medium (DMEM, Serotec, Oxford, UK) plus 10% fetal calf serum (FCS; Life Technologies Italia) and plated on 35 mm Petri dishes. After 24 h, the medium was supplemented with 10 μM Ara-C (Sigma-Aldrich). Medium was then changed with DMEM-FCS 10% plus 10 μM forskolin (Sigma-Aldrich) and 200 μg/mL bovine pituitary extract (BPE; Life Technologies Italia). Cells became confluent in 10 days. Immunopanning for final purification was carried out incubating the cells 30 min with mouse anti rat Thy1.1 antibody (Serotec, Italy), followed by 500 μL of baby rabbit complement (Cedarlane, Burlington, NJ, USA). Cell suspension (6 × 10^4^ cells) was seeded on 35 mm petri dishes, in presence of 2 µM forskolin. At the third in vitro passage, SCs were treated for 48 h with 4 μM forskolin, then used for different assays. SC purity (more than 98%) was tested with a specific antibody against glycoprotein P0 [30]. Phase-contrast images of DRG neurons and SCs in vitro cultures are provided in Supplementary Figure S2

### 2.3. Pharmacological Treatments

The desired concentration of each substance was achieved by dilution from stock into the culture medium. Substances used were: ALLO (Sigma-Aldrich), human recombinant brain-derived neurotrophic factor (BDNF; Millipore, Darmstadt, Germany) and cyclotraxin B (CYCLO; generous gift by Dr. Michel M.M. Verheij). ALLO 1 µM concentration was used according to our previous experiments [28] and in order to exclude any possible involvement of GABA-mediated endogenous activity. Differentiated SCs primary cultures were treated for the indicated time after overnight serum free condition, while DRG neurons’ primary culture was treated for the indicated time after 24 h N2-free condition. Conditioned experiments on DRG neurons were performed comparing DRG neurons treated with ALLO-exposed (24 h) SC’s conditioned medium (named CM + ALLO) with DRG neurons treated with vehicle-exposed (ethanol; 24 h) SC conditioned medium (named CM CTRL). Furthermore, in some experiments DRG neurons were treated with ALLO plus CYCLO-exposed (24 h) SC conditioned medium (named CM + ALLO + CYCLO).

### 2.4. RNA Extraction and qRT-PCR

RNA samples from DRG neurons and SCs cultures were extracted using Trizol^TM^ (Life Technologies Italia) according to the manufacturer’s protocol, and quantified with NanoDrop2000 (Thermo Fisher Scientific, Monza, Italy). Pure RNA was obtained after DNAse treatment with a specific kit (Sigma-Aldrich). One µg of RNA was reverse-transcribed to cDNA using iScript™ Reverse Transcription Supermix for RT-qPCR (Bio-Rad, Segrate, Milan, Italy). Primers were designed by PrimerBlast software (NIH, Bethesda, MD, USA). Primer sequences for PKCε and the housekeeper genes α-tubulin, 18s-rRNA and β2-microglobulin are reported in the Table 1. Ten ng of cDNA for each sample were used for Real Time PCR. qRT-PCR was performed by measuring the incorporation of EVA Green dye (Bio-Rad) with a CFX 96 Real Time System-C1000 touch thermal cycler (Bio-Rad). Data analysis was performed using the CFX Manager 2.0 software (Bio-Rad). The threshold cycle number (Ct) values of both the calibrator and the samples of interest were normalized to the geometric mean of Ct of the endogenous housekeeping genes. Data analysis was performed according to the Pfaff method and results are expressed as relative expression, normalized on the mean of housekeeper genes. As calibrator we used the RNA obtained from control samples. BDNF and trkB mRNA expression was analyzed by TaqMan qRT-PCR instrument (CFX384 real time system, Bio-Rad) using the iScript^TM^ one-step RT-PCR kit (Bio-Rad), as previously described [31]. Briefly, samples were run in 384 wells formats in triplicate as multiplexed reactions. Data were analyzed with the comparative threshold cycle (∆∆Ct) method using β-actin as reference gene. The primer efficiencies were experimentally set up for each couple of primers. Primers and probes for BDNF, trkB and β-actin (Eurofins MWG-Operon) are reported in Table 1.

### 2.5. Immunofluorescence (IFL)

Nerves were explanted and de-sheeted, then fixed in 4% paraformaldehyde (PFA, Sigma-Aldrich), included in OCT (Sakura, Leiden, The Netherlands) and cut in cross sections. For teased fibers, a slight digestion was performed incubating nerves fragments in collagenase IV for 45 min, before fixing in 4% PFA (Sigma-Aldrich). Cells were fixed in 4% PFA and processed for immunostaining. Primary antibodies used in these experiments were the following: rabbit anti PKCε 1:200 (Abcam), rabbit anti phospho S729 PKCε 1:200 (Abcam), mouse anti SMI31 1:500 (Biolegend, San Diego, CA, USA), mouse anti SMI32 1:500 (Biolegend), rabbit anti trkB 1:200 (Santa Cruz Biotechnology, Dallas, TX, USA) and fluoromyelin 1:150 (Thermo Fisher Scientific). After washing, slides and nerves were mounted using Vectashield^TM^ (Vector Laboratories, Burlingame, CA, USA) and nuclei stained with 4,6-diamidino-2-phenylindole (DAPI). Confocal microscopy was carried out using a Zeiss LSM 900 Airyscan 2 (Zeiss, Gottingen, Germany) and images were processed with Image Pro-Plus 6.0 (Media Cybernetics, Bethesda, MA, USA). Controls for specificity included a lack of primary antibody.

### 2.6. Western Blotting

Protein samples were extracted in lysis buffer (PBS, 1% Nonidet P-40 and 1 mM EDTA; all by Sigma-Aldrich) containing a cocktail of protease inhibitors (Sigma-Aldrich). Samples were heated for 20 min at 55 °C to denature secondary structures, then 15 µg were loaded onto an SDS-PAGE gel (Criterion TGX; Bio-Rad) and run at 200 V for 40 min in running buffer. Gels were electroblotted to PVDF membrane (GE Healthcare, Milan, Italy). Membranes were blocked with 10% not-fat dry milk (Bio-Rad) in TBS before incubation with the primary antibody against BDNF diluted in the blocking solution (1:500, Santa Cruz Biotechnology). Results were standardized using β-actin (1:10,000, Sigma-Aldrich) as reference. Membranes were incubated with appropriated HRP-conjugated secondary antibodies (Cell Signaling Technology Inc., Milan, Italy). Immunocomplexes were revealed by enhanced chemiluminescence (GE Healthcare), visualized using the Chemidoc MP Imaging System (Bio-Rad) and analyzed by the Image Lab software (Bio-Rad).

### 2.7. Quantitative Analysis of ALLO by Using Liquid Chromatography Tandem Mass Spectrometry Analysis (LC-MS/MS)

ALLO from medium and cells was extracted according to Caruso et al. [32] with minor modification. Briefly, the ^13^C-pregnenolone (PREG-20,21-^13^C_2_; 10 ng/sample), as internal standard, was added to the samples. Purification was performed using a C_18_ cartridges (Discovery DSC-18, 500 mg, Supelco, Milano, Italy). The steroid fraction was eluted with methanol (5 mL) and the organic residue was reconstituted with methanol:water (1:1) before the injection in a RP-C_18_ analytical column (Hypersil GOLD, Thermo Fisher Scientific Inc., Rodano, Italy; 3 μm, 100 mm × 3 mm ID). The high-performance liquid chromatograph (Surveyor LC Pump Plus, Thermo Fisher Scientific Inc.) was coupled to a linear ion trap mass spectrometer (LC/MS; LTQ, Fisher Scientific Co, Hampton, New Hampshire, USA). Atmospheric pressure chemical ionization source operating in the positive ion mode was used as ion source. ALLO was identified comparing both the retention time and the tandem mass (MS/MS) spectrum with that of the reference pure compound. The quantitative analyses were done monitoring specific ions (multiple reacting monitoring, MRM) selected in the MS/MS spectrum obtained by collision of precursor ions and by means of calibration curves, using the ^13^C-pregnenolone as internal standard.

### 2.8. ELISA Assay

Serum free medium were collected from SCs culture after 24 h of treatment with ALLO 1 μM, and concentrated using Amicon ultra centrifugal filter of 3 kDa (Millipore). ELISA was done following the manufacturer’s instruction (Raybiotech). BDNF concentration data were obtained by interpolating the quadratic standard curve.

### 2.9. Statistic Analysis

Data were statistically evaluated using the statistical package GraphPad Prism 6.00 (San Diego, CA, USA), with independent or paired two-tailed samples *t*-tests, one-way ANOVA followed by post hoc tests (see figure legends). All data were expressed as mean ± s.e.m. of the determinations performed, and significance was set at *p* < 0.05. Experiments were repeated at least three times. In pharmacological experiments, cell culture samples were allocated to groups randomly, organizing the treatments on multi-well device. Graphs were created with GraphPad Prism 6.00.

## 3. Results

### 3.1. PKCε is Constitutively Expressed in PNS Cells and Tissue

**Figure 1:**
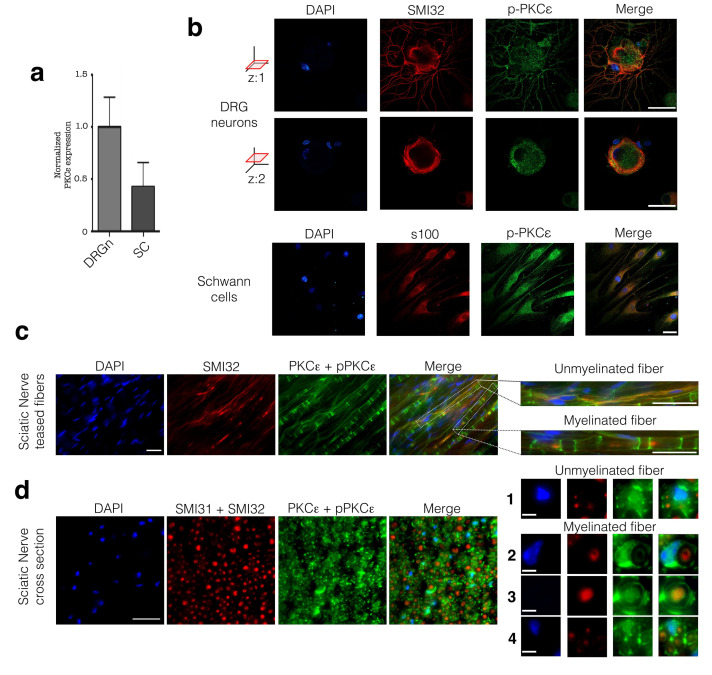
PKCε characterization in PNS. (**a**) qRT-PCR analysis confirmed the presence of PKCε in DRG neurons and SC primary cultures. Expression is normalized to the geometric mean of housekeeping genes and presented as fold change relative to the mean expression value for DRG neurons. (**b**) IFL analysis demonstrated the presence of the active/phosphorylated form of PKCε in DRG neuronal soma and arborization as well as in SCs primary cultures. Upper panels for DRG neurons (images refer to two *z*-axis optical sections): neurofilament marker SMI32 in red, *p*-PKCε immunopositivity in green and DAPI in blue; merge images show co-localization (in yellow). Lower panels for SC: s100 in red, *p*-PKCε in green and DAPI in blue; merge images show co-localization (in yellow). Bar: 30 μm. (**c**) Co-labeling of PKCε (green) and neurofilament marker SMI32 (red), coupled with morphology observation of myelinated and unmyelinated fibers, demonstrated that unmyelinated axons express PKCε (yellow signal and absence of myelin structure), and confirmed the presence of PKCε in SCs (green signal and typical myelin structure). (**d**) These observations were confirmed in coronal section of sciatic nerve (see also magnifications in right panels). Myelinating and unmyelinating SCs express PKCε in their cytosol and membrane (magnification 1 and 2 in green). Unmyelinated axons were immunopositive for PKCε (magnification 1). A subpopulation of myelinated axons were immunopositive for PKCε (magnification 3 in yellow), and in particular small diameter myelinated fibers (magnification 4 in yellow). DAPI in blue. Bars in panels c and d are 20 μm; magnifications in panels c, bars are 20 μm; magnifications 1, 2, 3, 4 in panel d, bars are 3 μm.

We first characterized PKCε expression in isolated DRG neurons and SCs in vitro. We found that PKCε is constitutively expressed by both cell types in vitro, observing higher PKCε gene expression in cultured rat DRG neurons, than in SCs (Figure 1a). IFL images confirmed that the PKCε protein was present both in DRG neurons (Figure 1b, upper panels; images in two *z*-axis optical sections) and SCs (Figure 1b, lower panels); specificity was assessed by co-labeling with SMI32 (for high-density NF) and s100, markers of DRG neurons and SC, respectively. These images showed that the phosphorylated form of PKCε is evenly distributed throughout the cytoplasm (Figure 1b), both in neuronal soma and SC, as well in neuronal arborization. Moreover, IFL showed localization of PKCε along unmyelinated as well as myelinated fibers. Double-labelling of teased fibers with PKCε and SMI32 showed the presence of phosphorylated and non-phosphorylated PKCε in unmyelinated axons (Figure 1c magnification upper panel), and in surrounding non-myelinating SCs (Figure 1c; magnification lower panel). Furthermore, myelinating SCs were immunopositive for PKCε (Figure 1c, magnification lower panel). These observations were further confirmed in cross sections of the sciatic nerve, in which PKCε is clearly present in unmyelinated axons and in surrounding SCs (Figure 1d, magnification 1), as well as in myelinating SCs (Figure 1d, magnification 2, 3, 4). Cross sections allowed us to detect a subset of myelinated fibers with PKCε also inside axons (Figure 1c, magnification 3 and 4), and in particular in small diameter fibers (Figure 1d, magnification 4).

### 3.2. Neuronal PKCε Is Regulated by an SC’s Humoral Factor

**Figure 2:**
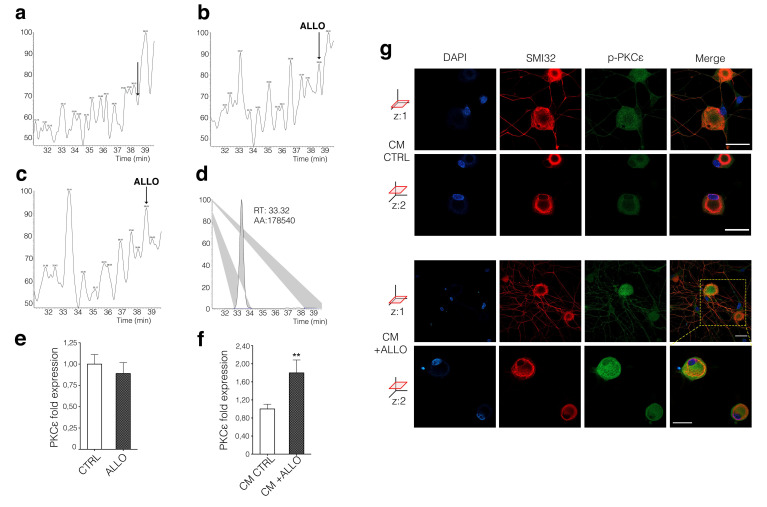
ALLO is released by SCs and modulates neuronal PKCε. LC-MS/MS analysis of SCs cytosol and KREBS buffer from 6 h-cultured SCs demonstrated the presence of ALLO. Multiple reaction monitoring (MRM) traces of (**a**) blank sample (**b**) cytosolic fraction (**c**) medium fraction and (**d**) internal standard, ^13^C-pregnenolone (PREG-20, 21-^13^C_2_; 10 ng/sample). Arrows indicate relative retention time of ALLO (RT: 31.01–39.63; SM: 11G; *m/z* = 158.50 − 159.50 + 172.50 − 173.50 + 212.50 − 213.50 + 226.50 − 227.50; F:ITMS + cAPCI corona sid = 15.00; w Full ms2 [80.00–330]). (**e**) DRG neurons directly treated with ALLO (1 μM) did not show PKCε expression modulation (CTRL *n* = 15, ALLO *n* = 8; *p* = 0.6284. The experiment was repeated four times. Data are mean ± s.e.m.). (**f**) PKCε expression was affected in DRG neurons by ALLO-treated SC’s conditioned medium (CM + ALLO), showing an upregulation in qRT-PCR, compared to ethanol-treated SC’s conditioned medium (CM CTRL), (*p =* 0.0040, *t* = 3.248, CM CTRL *n* = 15, CM + ALLO *n* = 7 unpaired Student *t*-test. The experiment was repeated three times. Data are mean ± s.e.m.; ** *p* < 0.01) (**g**) IFL analysis confirmed the increase in PKCε signal intensity in both soma (*z*: two axes optical section) and arborization (*z*: one axis optical section) of DRG neurons treated with CM + ALLO, compared with controls (CM CTRL), respectively; neurofilament marker SMI32 in red, *p*-PKCε in green, DAPI in blue, merge co-localization in yellow. Bar: 30 μm.

It is know that SCs synthesize the progesterone metabolite ALLO, one of the most important mediator of neuron-glia interaction in peripheral nerves [6,7,8,9]. By means of HPLC-MS analysis, we show here unequivocally the presence of ALLO 0.68 pg/μL (2.13 ± 0.95 nM) in the cytosol of SCs (Figure 2b). ALLO 0.23 pg/μL (0.72 ± 0.43 nM) was also detected in Krebs-Ringer buffer exposed to cultured SCs for 6 h, under a basal culture condition (Figure 2c). This finding confirmed that SCs release ALLO physiologically. Based on this observation, we further investigated whether ALLO might modulate PKCε expression in DRG neurons in vitro, confirming previous preliminary data already published [28]. After 24 h treatment with ALLO at 1 μM, the PKCε expression in DRG neurons was unaltered (Figure 2e). However, when the conditioned medium (CM) harvested from SCs, treated for 24 h with ALLO 1 μM, was applied to DRG neuronal cultures for another 24 h, the PKCε gene expression was significantly (*p* < 0.01) increased (Figure 2f), suggesting that a humoral mediator of glia-to-neuron cross-talk was able to regulate PKCε. IFL analysis corroborated these data, showing that PKCε protein was increased in DRG neurons exposed to the CM from ALLO-treated SCs (Figure 2g). Images from two *z*-axis optical sections, indeed, showed *p*-PKCε immunopositivity increase (green) both in neuronal soma and arborization (labeled with neurofilament marker SMI32 in red; Figure 2g).

### 3.3. ALLO Regulates the Production and Release of BDNF by SCs

**Figure 3:**
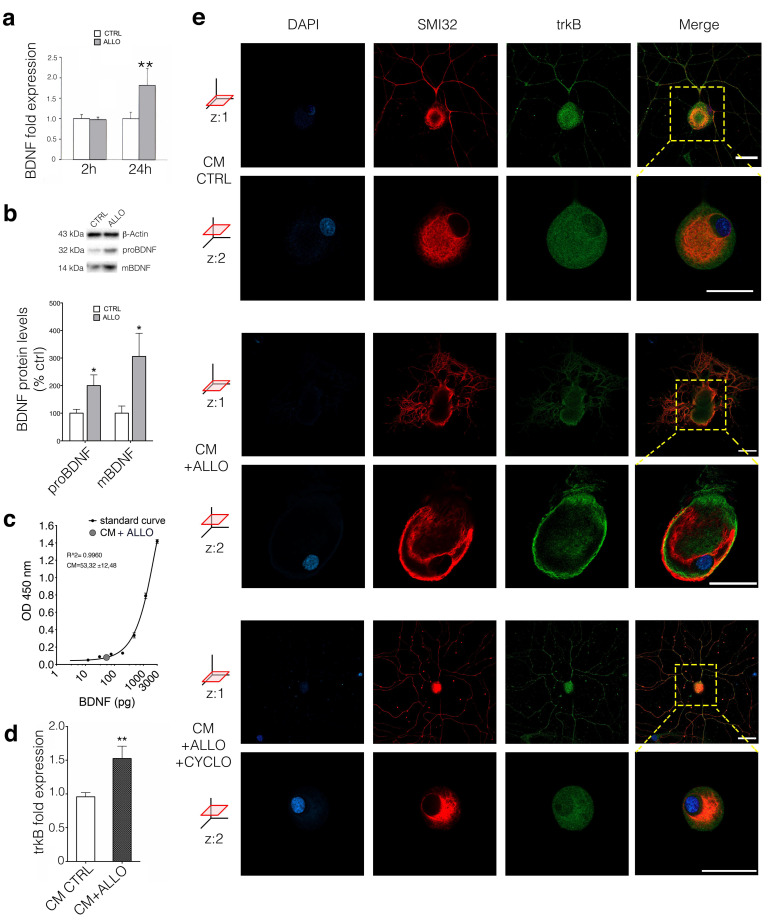
BDNF is the neuron-glia mediator responsible of PKCε modulation in DRG neurons. (**a**) SCs treated with ALLO 1 μM for 24 h showed BDNF expression upregulation (*p* = 0.0034, *t* = 3.52, *n* = 14 unpaired Student *t*-test; the experiment was repeated four times; data are mean ± s.e.m.; ** *p* < 0.01) (**b**) Western blot analysis that demonstrated upregulation of proBDNF (*p* = 0.033, *t* = 2.42, *n* = 12, unpaired Student *t*-test) and mature BDNF (mBDNF; *p* = 0.046, *t* = 2.20, *n* = 13, unpaired Student *t*-test; the experiment was repeated three times; data are mean ± s.e.m. of protein levels; * *p* < 0.05). (**c**) ELISA assay on conditioned medium (CM + ALLO) from SCs (SCs treated 24-h with ALLO 1 μM) showed a BDNF concentration of 1.7 ± 0.39 pM (*n* = 5 ± s.d.). (**d**) DRG neurons treated with SCs conditioned medium (CM + ALLO) showed an upregulation of trkB receptor expression (*p* = 0.0012, *t* = 3.556, unpaired t test; CM CTRL *n* = 21, CM + ALLO *n* = 12; the experiment was repeated four times; data are mean ± s.e.m.; ** *p* < 0.01). (**e**) Confocal images (at two *z*-axis optical sections) showed trkB translocation in cell membrane (*z*:2) after ALLO-treated SC conditioned medium (CM + ALLO), compared to control treatment (CM CTRL). The CM + ALLO added with the specific trkB antagonist CYCLO (CM + ALLO + CYCLO) did not induce any trkB translocation in neuronal membrane; neurofilament marker SMI32 in red, trkB in green, DAPI in blue, merge co-localization in yellow. Bar: 30 μm.

To identify the mediator responsible for the CM-induced effect in DRG neurons, we focused our attention on the growth factor BDNF, which was previously shown to be released by activated SCs [33,34]. Exposure of SC cultures to ALLO 1 μM showed a significant elevation of BDNF expression after 24 but not 2 h (Figure 3a; *p* < 0.05). Accordingly, both precursors (proBDNF) and mature (mBDNF) BDNF protein levels increased significantly (Figure 3b; *p* < 0.05). The level of BDNF in 24-h conditioned medium from SCs was measured by ELISA assay, resulting in 52.32 pg/mL (1.7 ± 0.39 pM) concentration (Figure 3c). Treating cultured DRG neurons for 24 h with SCs’ CM + ALLO we found an upregulation of the high affinity BDNF receptor trkB, corroborating the hypothesis of a BDNF-related mechanism (Figure 3d; *p* < 0.01). IFL confirmed trkB activation (in green) in DRG neurons (positive for the neurofilament marker SMI32 in red) after CM + ALLO treatment, with evidence of receptor translocation to the cell membrane detected at *z*: two axes optical section (Figure 3e). Furthermore, as shown in Figure 3e (at two *z*-axis optical sections) SCs’ CM effect on trkB activation was completely blocked by the co-treatment with the specific trkB antagonist cyclotraxin-B (CYCLO) [35].

### 3.4. BDNF Regulates PKCε in DRG Neurons Via trkB Activation

**Figure 4:**
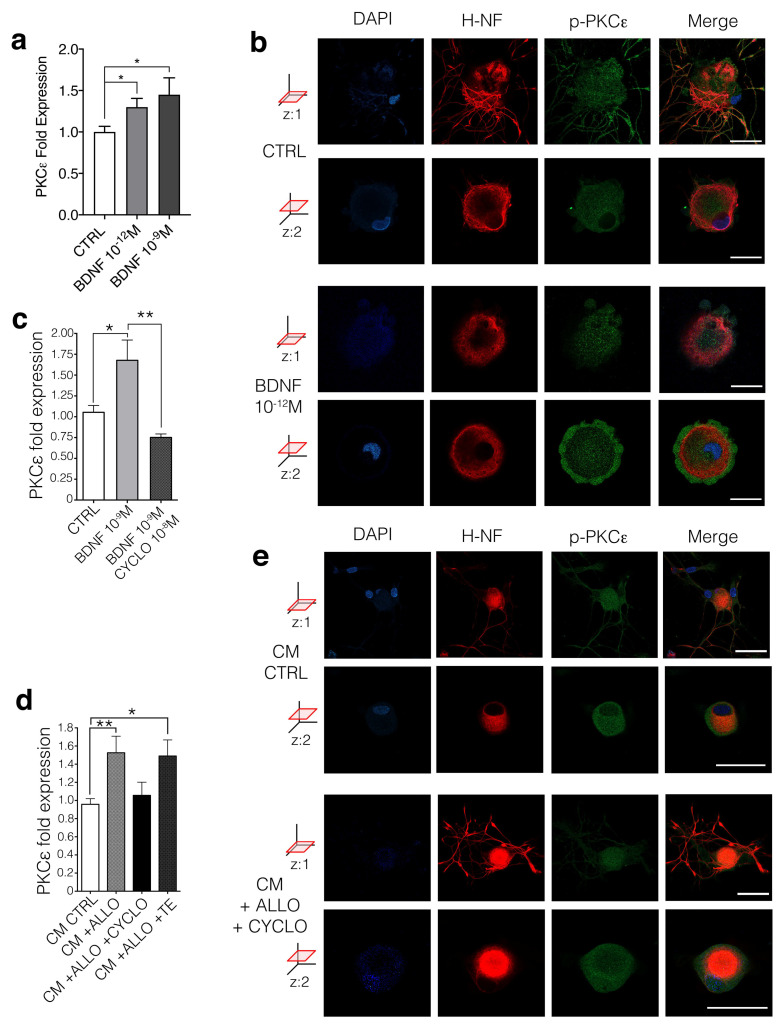
Glial BDNF modulates PNCε expression via trkB and induces its activation. (**a**) ALLO-treated SCs’ CM effect has been mimicked by exogenous human recombinant BDNF treatment (one-way ANOVA, *p* = 0.0278, F = 3.954; Brown-Forsythe test *p* = 0.0314, F (2.37) = 3.804; *n* = 40; Unpaired *t*-test CTRL vs. BDNF 10^−9^ M, *p* = 0.0186, *t* = 2.517, df = 25. Unpaired t test CTRL vs. BDNF 10^−12^ M, *p* = 0.0105, *t* = 2.775, df = 24. The experiment was repeated four times. Data are mean ± s.e.m.; * *p* < 0.05). (**b**) Confocal images (at two *z*-axis optical sections) showed that exogenous BDNF (1 pM) treatment caused a fast PKCε reorganization in DRG neurons, moving active-*p*-PKCε in the membrane (z:2). Neurofilament marker SMI32 in red, *p*-PKCε in green, DAPI in blue, merge co-localization in yellow. Bar: 30 μm. (**c**) PKCε modulation mediated by exogenous BDNF was blocked by specific trkB antagonist CYCLO (one-way ANOVA *p* = 0.0016, F = 7.972; Brown-Forsthe test *p* = 0.0017, F (2.31) = 7.909; *n* = 34; Tukey’s multiple comparison test CTRL vs. BDNF 10^−9^ M *p* = 0.0115, q = 4.362; BDNF 10^−9^ M vs. BDNF 10^−9^ M + CYCLO *p* = 0.0023, q = 5.240. The experiment was repeated three times. Data are mean ± s.e.m.; * *p* < 0.05, ** *p* < 0.01). (**d**) CYCLO treatment is also able to block CM + ALLO action on DRG neurons (one-way ANOVA *p* = 0.0024, Bartlett’s test *p* = 0.0252; *n* = 49; Tukey’s multiple comparison test CM CTRL vs. CM + ALLO *p* = 0.0054; CM CTRL vs. CM + ALLO + CYCLO *p* = 0.9507; CM CTRL vs. CM + ALLO +TE *p* = 0.0307. The experiment was repeated three times. Data are mean ± s.e.m.; * *p* < 0.05, ** *p* < 0.01). (**e**) IFL confirmed that CYCLO 10 nM blocked the effect of SCs’ CM + ALLO increase in *p*-PKCε immunopositivity (see Figure 2g), lowering back to the levels detected in control (CM CTRL). Neurofilament marker SMI32 in red, *p*-PKCε in green, DAPI in blue, merge co-localization in yellow. Bar: 30 μm.

To replicate the effects of CM + ALLO on PKCε levels in DRG neurons, we treated these cells with human recombinant BDNF, at 1 pM and 1 nM, respectively. Both BDNF concentrations significantly upregulated PKCε gene expression (*p* < 0.05) after 24-h exposure (Figure 4a). IFL analysis highlighted the additional effect of BDNF (Figure 4d), showing translocation of the phosphorylated form of *p*-PKCε (green; at *z*: two axes optical section) to the membrane of DRG neurons (positive for SMI32 in red), treated transiently (30 s) with BDNF 1 pM (Figure 4b). Then, we tested whether BDNF was affecting neuronal PKCε expression via its classic receptor trkB. In support of a specific role of the trkB receptor, DRG neurons were co-treated with BDNF 1 nM and the trkB specific antagonist CYCLO. qRT-PCR analysis showed that CYCLO 10 nM was able to completely block PKCε upregulation induced by BDNF (Figure 4c). Equally, CYCLO 10 nM completely blocked the effect of SCs’ CM on PKCε expression (Figure 4d), as well as the *p*-PKCε rise in immunopositivity (Figure 4e, compared to Figure 2g), indicating that the antagonist was blocking the effect of endogenous BDNF present in the CM. As a control, the inactive form of CYCLO (TE) did not reverse the CM effect, confirming the specificity of the trkB antagonist (Figure 4c). Overall, we demonstrated that secondary to ALLO release, SCs increased BDNF, which upon secretion led to trkB-mediated upregulation of PKCε in DRG neurons.

## 4. Discussion

**Figure 5:**
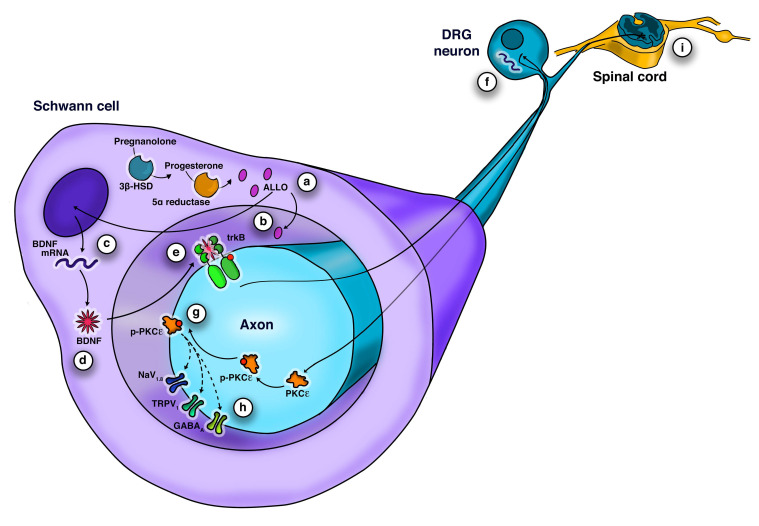
Schematic representation of neuron-glia cross talk in peripheral fibers. (**a**) SCs possess the enzymatic machinery to synthetize ALLO [9], (**b**) and tonically release it in vitro. (**c**) ALLO increases BDNF expression, and (**d**) its protein levels in SCs. (**e**) BDNF is released by SCs and activates neuronal trkB. (**f**) TrkB mediates the upregulation of PKCε expression in DRG neurons (**g**) and a simultaneous PKCε activation consisting in its phosphorylation and translocation to neuronal membrane. (**h**) PKCε is renown to increase neuronal excitability via the phosphorylation of several ion channels, likely promoting hyperalgesic priming of peripheral fibers [19]. (**i**) leading to the modulation of noxious stimuli to the CNS.

Our experiments demonstrated that ALLO promoted SC-dependent activation of a signaling cascade, involving BDNF release and neuronal PKCε upregulation. In detail, we found that SCs tonically release ALLO, which in turn upregulates the synthesis and release of BDNF in an autocrine fashion. Subsequently, BDNF controls PKCε in DRG sensory neurons, via trkB activation in paracrine manner (Figure 5).

This line of evidence is consistent with the predominance of the local interaction, in the PNS, between SCs and peripheral sensory neurons. Beyond their myelinating activity, SCs exert a crucial role on neuronal functions, strengthening their importance in glia-to-neuron crosstalk. Although these effects occur in the microdomain of SCs-axon, they may reflect on the neuronal soma, likely DRG neurons, given that the retrograde transport of molecules and proteins along axons was widely demonstrated in the PNS [36,37]. Moreover, it was proven that SCs-derived neurotrophic factors are transported anterogradely as well as retrogradely within peripheral neurons [38].

We found that SCs are capable of ALLO synthesis and its tonic release under physiological conditions. Notably, ALLO synthesized by SCs might attain efficient concentration in the narrow space between SC membrane and axon, both in myelinated fibers (i.e., in the periaxonal/adaxonal space) and in unmyelinated fibers (i.e., Remak bundles). The observation that ALLO serves as an autocrine factor in SCs is not new, since it was already demonstrated that ALLO enhances the glutamic acid decarboxylase (GAD) expression/activity and subsequently GABA synthesis in SCs [13,15]. Interestingly, ALLO has been proposed as a possible pharmacological treatment for nerve degenerative diseases [10,11,14,15,39] and hyperalgesia [39]. Nerve regeneration, occurring after peripheral nerve damage, is generally associated with hypersensitivity and possible development of chronic pathological pain states [40]. A similar phenomenon has been observed for neurotrophins, which proved to be potent modulators of neuroregeneration and neuronal plasticity [41], within a concomitant increase in excitability. In the CNS, BDNF has been reported to enhance neuronal excitability [42], promoting neurotransmitter release [43], to phosphorylate specific glutamate receptor subunits [44,45] and to modulate ion channel conductance [46]. Similarly, in the PNS, BDNF expression in DRGs is markedly increased by injury or inflammation [47,48,49] whereas trkB activation promotes synaptic plasticity in the dorsal horn [47].

The ALLO-induced BDNF synthesis and release have been previously demonstrated in the CNS [50]. Herein, we suggest that in the PNS, the increase of ALLO and consequent rise of BDNF local release might entail a dualistic effect on nerves. This promotes regeneration and acute control of pain, likely triggering the activation of an intracellular cascade that induces a long-term sensitization of neurons, thus priming peripheral nociceptors [19,20].

BDNF as well as trkB receptor expression in a distinct subset of peripheral sensory neurons have been well characterized. Indeed, by means of electrophysiological techniques [51,52] and single-cell sequencing approaches [52,53,54], their expression was highlighted in low threshold mechanosensitive A delta fibers [55], and in a small subset of C-nociceptors [56,57,58]. In accordance, trkB specific expression in these peripheral nociceptors provides a pathway through which SC derived BDNF can regulate neuropathic pain. Likewise, we found that PKCε is expressed in thinly myelinated fibers (low threshold mechanosensitive A delta) and unmyelinated fibers (C-nociceptors), supporting the interaction between peripheral trkB and PKCε. Generally, the A delta and C fibers increase in excitability leads to allodynia and hyperalgesia, respectively.

PKCε presence in sensory neurons is consistent with previous observation in vivo, showing its activation in rat lumbar DRG neurons during inflammatory and neuropathic pain [59]. Interestingly, PKCε activation in DRG sensory neurons is likely to be associated with its capacity for ion channel phosphorylation. PKCε increases the open probability of TRPV1 [21,22], decreases GABA_A_-R membrane trafficking [23,24] and upregulates Na_v_1.8 expression [26]. In particular, it should be highlighted that PKCε promotes GABA_A_-R desensitization. Namely, PKCε phosphorylates the γ2 subunit of GABA_A_-R, reducing its responsivity to specific allosteric agonists [27]. Moreover, PKCε regulates GABA_A_-R trafficking, decreasing its cell surface expression and GABA currents [24]. Such a specific interaction between PKCε and GABA_A_-R might partially explain the non-canonical role of ALLO on pain modulation. ALLO can indeed potentiate GABA_A_-R currents acutely, acting as an allosteric modulator [39], but in chronic conditions it could lead to the release of BDNF and thus PKCε activation, eliciting an opposite effect and inducing hypersensitivity.

In conclusion, we provide consistent evidence that SCs are a peripheral local source for the neuroactive steroid ALLO, which leads to BDNF release and paracrine PKCε upregulation, via neuronal trkB activation. However, further studies are required to characterize the physio-pathological responses occurring in DRG sensory neurons following PKCε activation. We suggest that further characterization of these molecular mechanisms of glia-to-neuron interaction may foster our understanding of neuropathic pain etiology, helping to identify novel targets for pharmacological treatments.

## Figures and Tables

**Table 1:** Sequences of primers used in the qRT-PCR.

Primer Name	Forward Primer 5′–3′	Reverse Primer 5′–3′	Probe (When Applicable)
PKCε	CCCCTTGTGACCAGGAACTA	AGCTGGCCATCAGTAGACGA	
BDNF	AAGTCTGCATTACATTCCTCGA	GTTTTCTGAAAGAGGGACAGTTTAT	GATCAGGTCAGACAAGTCAAGG
trkB	GTGGATTCCGGCTTAAAGTTTG	GATCAGGTCAGACAAGTCAAGG	CCTGCGGCACATCAATTTCACTCG
α-tubulin	TCGCGCTGTAAGAAGCAACACC	ATGGAGATGCACTCACGATGGT	
18s	CTGCCCTATCAACTTTCGATGGTAG	CCGTTTCTCAGGCTCCCTCTC	
β2-microglobulin	ACATACGCCTGCAGAGTTAAGC	TGCTTGATCACATGTCTCGATCCC	
β-actin	CACTTTCTACAATGAGCTGCG	CTGGATGGCTACGTACATGG	TCTGGGTCATCTTTTCACGGTTGGC

## Supplementary Materials

The following are available online at https://www.mdpi.com/2073-4409/9/8/1874/s1, Supplementary Figure S1: uncropped immunoblot related to the expression levels of proBDNF (32 kDa), mBDNF (14 kDa) and b-Actin (43 kDa) measured in the cell lysate of SCs treated with ALLO, presented in Figure 3; C = control; A = ALLO, Supplementary Figure S2: phase-contrast images of DRG neurons and SCs in vitro cultures; bars: 30 μm, Supplementary Figure S3: CSLM *z* stack images comparing medium diameter DRG neurons (*z* = 9 μm) vs. small diameter DRG neurons (*z* = 4 μm); bars: 10 μm.
