# Supplementary figures and images for: Schwann Cell Autocrine and Paracrine Regulatory Mechanisms, Mediated by Allopregnanolone and BDNF, Modulate PKCε in Peripheral Sensory Neurons
Source: Cells. 2020 Aug 11;9(8):1874. doi: 10.3390/cells9081874 (PMC7465687; doi:10.3390/cells9081874)

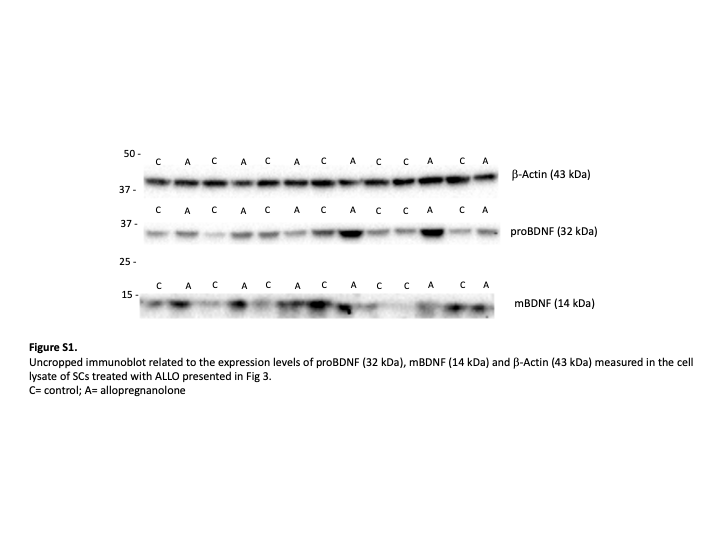

Supplement: Supplementary file 1 [file cells-09-01874-s001.zip › SupplementaryFigures/FIGURE S1.tiff]

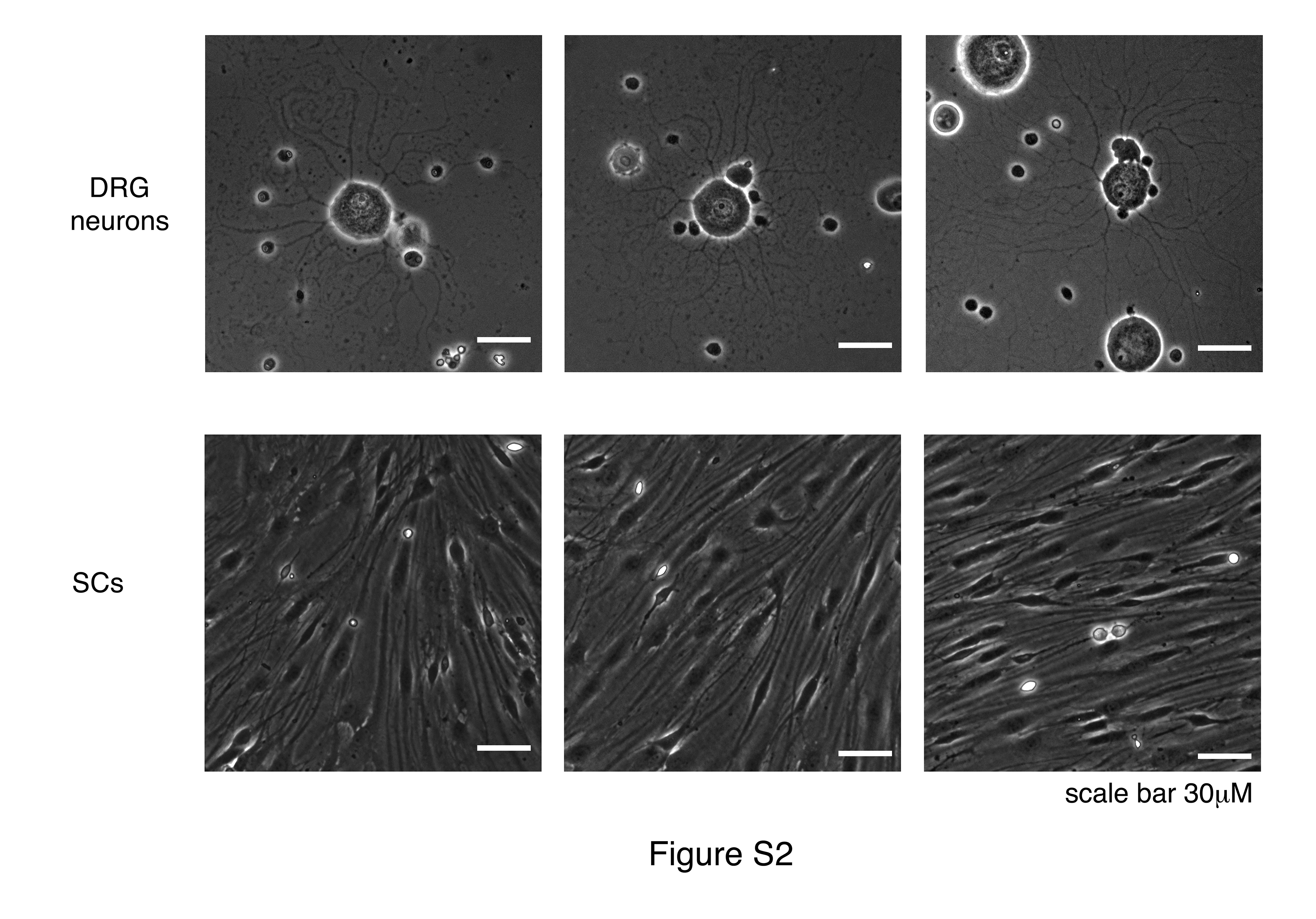

Supplement: Supplementary file 1 [file cells-09-01874-s001.zip › SupplementaryFigures/FIGURE S2.tif]

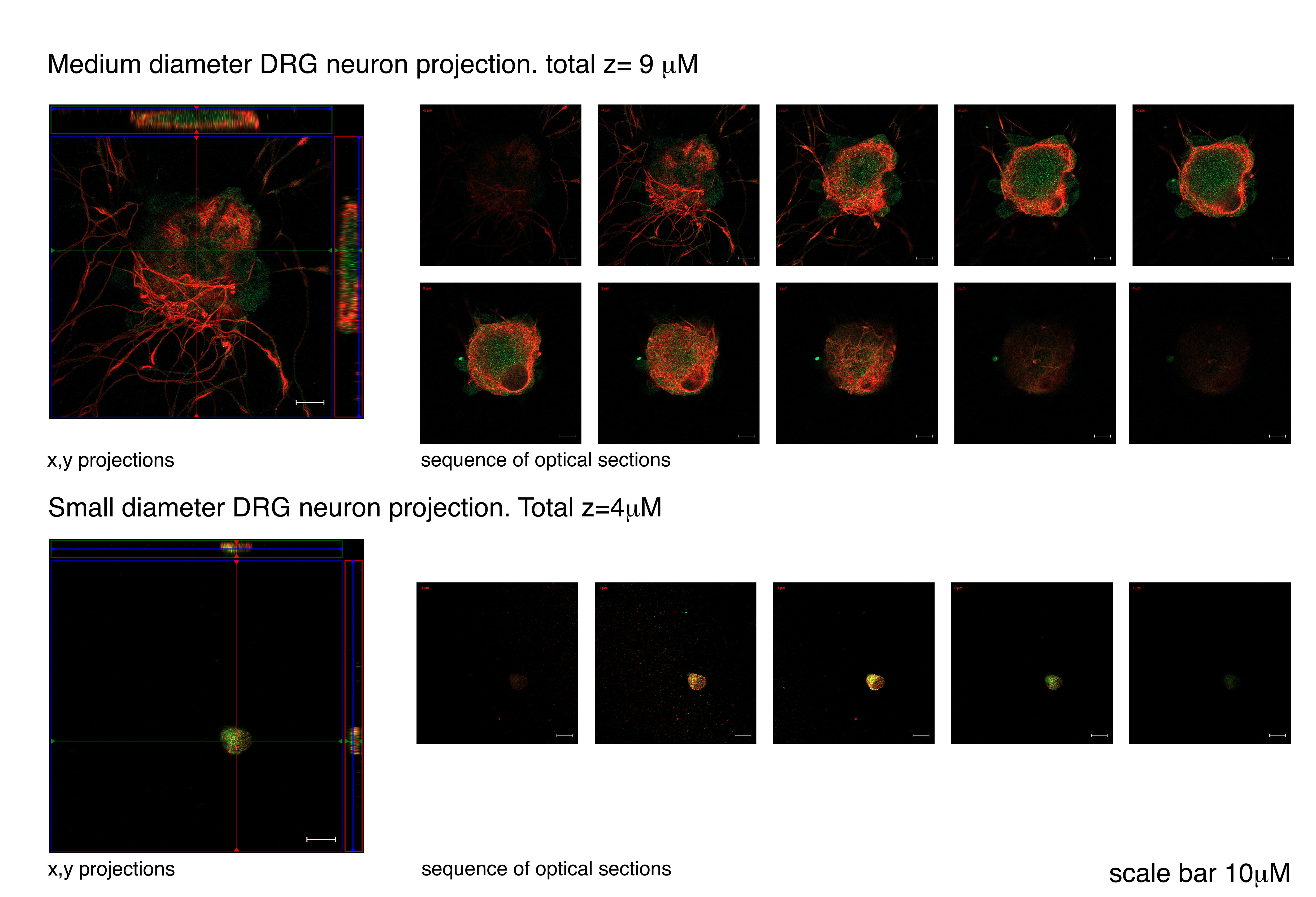

Supplement: Supplementary file 1 [file cells-09-01874-s001.zip › SupplementaryFigures/FIGURE S3.tif]
